# The role of epigenetic changes in the pathology and treatment of inherited retinal diseases

**DOI:** 10.3389/fcell.2023.1224078

**Published:** 2023-08-04

**Authors:** Annie L. Miller, Rebekah E. James, Alan R. Harvey, Dragana Trifunović, Livia S. Carvalho

**Affiliations:** ^1^ Centre for Ophthalmology and Visual Science, The University of Western Australia, Crawley, WA, Australia; ^2^ Retinal Genomics and Therapy Laboratory, Lions Eye Institute, Nedlands, WA, Australia; ^3^ School of Human Sciences, The University of Western Australia, Crawley, WA, Australia; ^4^ Perron Institute for Neurological and Translational Science, Nedlands, WA, Australia; ^5^ Institute for Ophthalmic Research, Tubingen University, Tübingen, Germany; ^6^ Department of Optometry and Vision Sciences, Faculty of Medicine, Dentistry and Health Sciences, The University of Melbourne, Melbourne, VIC, Australia

**Keywords:** inherited retinal disease, epigenetic changes, DNA methylation, histone methylation, histone acetylation, poly(ADP-ribosyl)ation

## Abstract

Elucidation of the cellular changes that occur in degenerating photoreceptors of people with inherited retinal diseases (IRDs) has been a focus for many research teams, leading to numerous theories on how these changes affect the cell death process. What is clearly emerging from these studies is that there are common denominators across multiple models of IRD, regardless of the underlying genetic mutation. These common markers could open avenues for broad neuroprotective therapeutics to prevent photoreceptor loss and preserve functional vision. In recent years, the role of epigenetic modifications contributing to the pathology of IRDs has been a particular point of interest, due to many studies noting changes in these epigenetic modifications, which coincide with photoreceptor cell death. This review will discuss the two broad categories of epigenetic changes, DNA methylation and histone modifications, that have received particular attention in IRD models. We will review the altered epigenetic regulatory events that are believed to contribute to cell death in IRDs and discuss the therapeutic potential of targeting these alterations.

## 1 Introduction

Inherited retinal diseases (IRDs) are a genetically and phenotypically diverse group of blinding diseases that can result in photoreceptor death, dysfunction, or developmental delay (Berger et al., 2010). Collectively, these diseases affect 1:2000 people worldwide and pose a significant socioeconomic problem due to healthcare costs, reduced workplace participation and an increased requirement for carer assistance (Berger et al., 2010; Galvin et al., 2020). However, treatments available for IRD are limited; only people with a mutation in one particular gene, *RPE65*, can receive the FDA-approved gene therapy drug Luxturna, leaving a critical gap in patient care (Maguire et al., 2021). Mutations in over 270 genes have been associated with IRD to date, and more are being discovered (Center DSTUoTHS, 2020). Due to this genetic heterogeneity, many researchers have investigated common targets that are independent of the underlying genetic mutations, with the aim of developing neuroprotective therapies that can treat a broader population of IRD patients. Such studies often focus on understanding the precise cell death mechanisms that lead to photoreceptor death. There is extensive debate in the field, with conflicting reports on whether apoptotic or non-apoptotic cell death mechanisms, or somewhere “in-between”, are the predominant cause of photoreceptor loss (Brunet et al., 2022). A seminal study by Arango-Gonzalez et al. (2014) identified a common non-apoptotic cell death pathway that was dysregulated in ten mouse models of IRD, with many of the components of this pathway linked to epigenetic regulation (Arango-Gonzalez et al., 2014). In recent years there has been increased research in this area, strengthening links between epigenomic modifications and cell death in IRD. This review will outline the current understanding of the association of two types of epigenetic modification, DNA methylation and histone modifications, with IRD pathology.

## 2 DNA methylation

**FIGURE 1:**
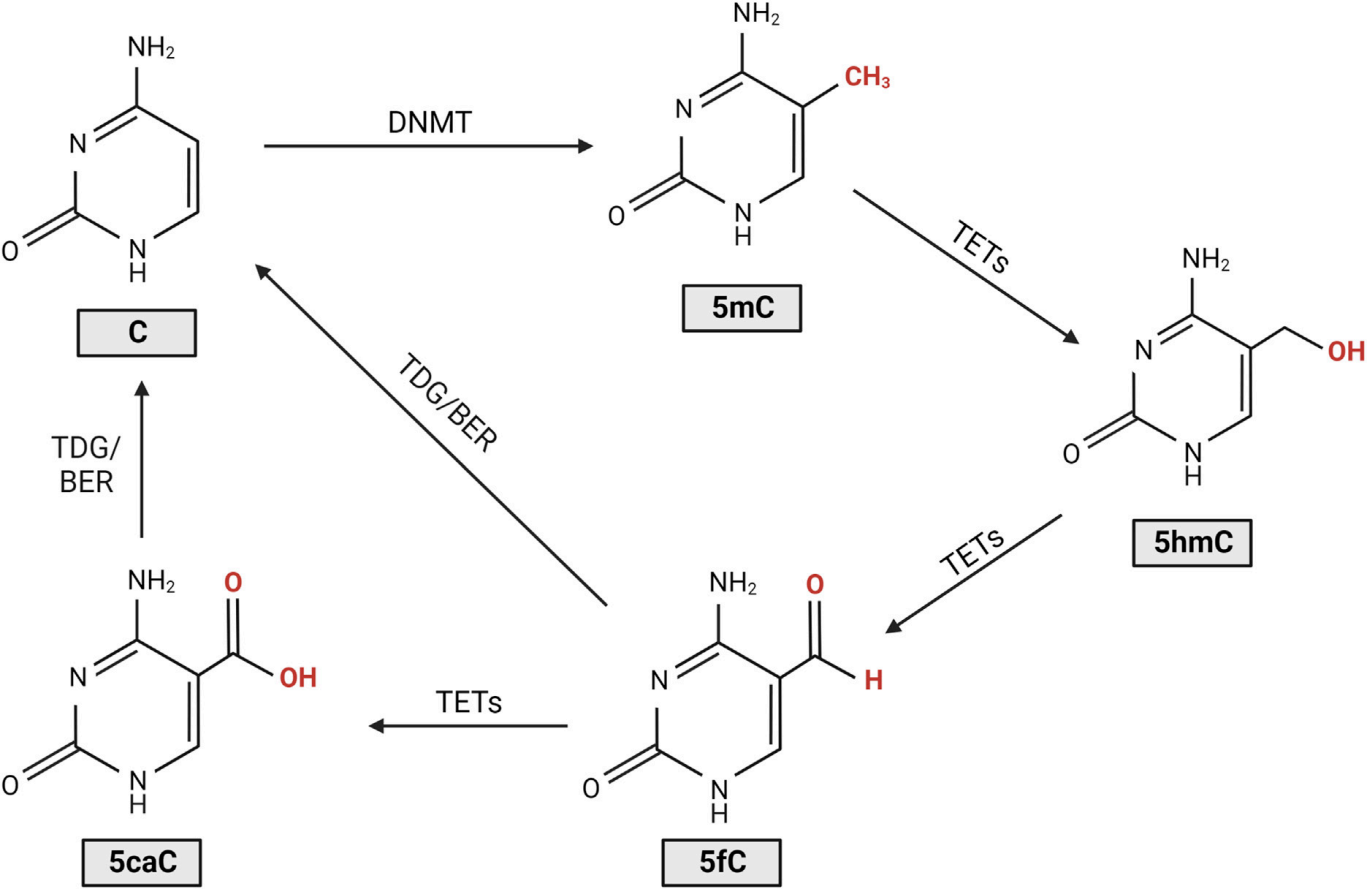
Cytosine DNA methylation and demethylation. DNA methyltransferases (DNMTs) introduce a methyl group to the cytosine (C), forming 5-methylcytosine (5mC). Ten-eleven translocase (TET) enzymes then regulate the oxidation of 5mC to 5-hydroxymethylcytosine (5hmC), 5-formylcytosine (5fC), and 5-carboxylcytosine (5caC). Following oxidation to 5fC or 5caC, restoration of the molecule to cytosine is modulated by thymine DNA glycosylase (TDG), an essential component of the base excision repair (BER) pathway (Moore et al., 2013; Rasmussen and Helin, 2016).

DNA methylation is a heritable genetic mark essential in multiple developmental processes such as genomic imprinting, X-chromosome inactivation and suppression of repetitive element transcription (Jin et al., 2011). DNA methylation functions by recruiting proteins involved in gene repression while also having a role in blocking DNA transcription factors (Moore et al., 2013). In eukaryotes, DNA methylation most often involves the addition of a methyl group to the C5 position of cytosine, forming 5-methylcytosine (5mC) (Moore et al., 2013). Other forms of DNA methylation exist, namely N6-methyladenine and N4-methylcytosine; however, their role in eukaryotes is far less clear, and thus they will not be a focus of this review (Xiao et al., 2018; Rodriguez et al., 2022). The level of methylation and demethylation of DNA is modulated by DNA methyltransferases (DNMTs) and ten-eleven translocase (TET) enzymes (Figure 1) (Moore et al., 2013; Rasmussen and Helin, 2016). DNMTs catalyse DNA methylation by transferring a methyl group to the fifth carbon of a cytosine to form 5mC (Moore et al., 2013). TET enzymes regulate the oxidation of 5mC to 5-hydroxymethylcytosine (5hmC), which can be further oxidised to form 5-formylcytosine (5fC) and 5-carboxylcytosine (5caC), leading to DNA demethylation (Moore et al., 2013). After oxidation to 5fC or 5caC, restoration of the molecule to a cytosine is modulated by thymine DNA glycosylase (TDG), which is an essential component of the base excision repair (BER) pathway (Moore et al., 2013). Changes to the proportion of oxidised cytosines and DNMTs are involved in multiple pathologies such as cancer and are thought to potentially contribute to photoreceptor degeneration in models of IRD (Wahlin et al., 2013; Farinelli et al., 2014; Locke et al., 2019). 5mC and 5hmC are the best understood of the cytosine derivatives and are thought to be the most biologically relevant thus far. This review will focus on studies that involve their dysregulation in IRDs.

**FIGURE 2:**
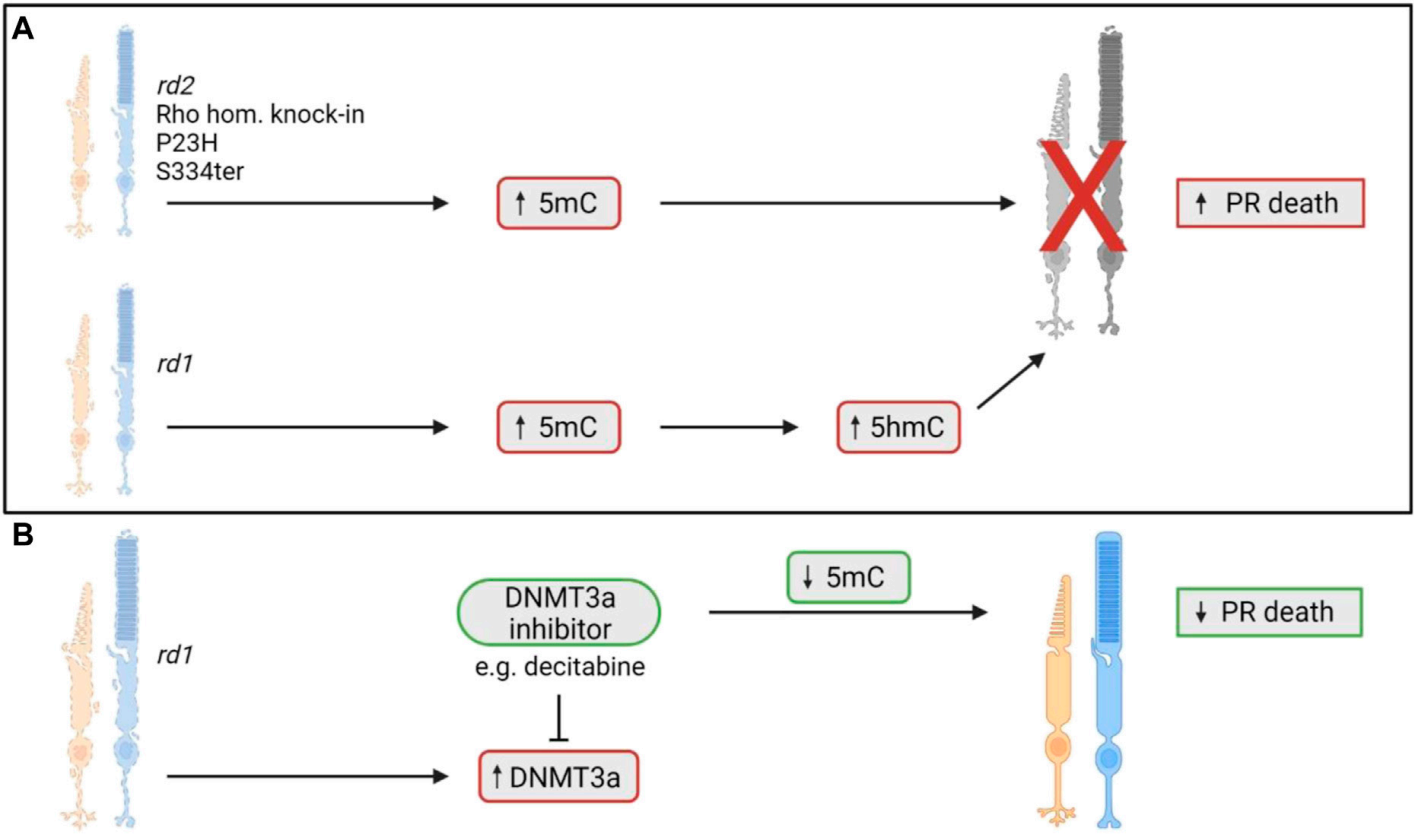
DNA methylation changes involved in photoreceptor death in models of IRD. **(A)** Previous studies have shown an upregulation of the demethylated cytosine molecule, 5-methylcytosine (5mC), in five models of IRD (Wahlin et al., 2013; Farinelli et al., 2014). The 5mC upregulation coincides with the photoreceptor degeneration found in each model (Wahlin et al., 2013; Farinelli et al., 2014). In the *rd1* mouse model only, increased levels of the 5-hydroxymethylcytosine (5hmC) molecule was noted as well (Wahlin et al., 2013). 5hmC is an oxidised form of 5mC, with increased levels coinciding with photoreceptor death (Wahlin et al., 2013). **(B)** In the *rd1* mouse, there was increased expression of DNMT3a, an enzyme responsible for the demethylation of cytosine to form 5mC. When *rd1* retinal explants were treated with the DNMT3a inhibitor, decitabine, they noted a decrease in 5mC positive cells and a reduction in photoreceptor cell death (Farinelli et al., 2014). PR = photoreceptor.

Wahlin et al. (2013) first reported aberrant DNA methylation levels in the *rd1* mouse model of retinitis pigmentosa (RP), a widely used model that displays rapid rod photoreceptor loss that peaks between postnatal days 12–14 (P12-14; for a summary of all preclinical models discussed in this review, refer to Supplementary Table S1) (Keeler, 1924; LaVail and Sidman, 1974; Portera-Cailliau et al., 1994; Wahlin et al., 2013). At timepoints corresponding to this peak of rod cell death, both rod and cone photoreceptors in the *rd1* retina were found to possess increased immunoreactivity for 5mC and 5hmC compared to wildtype controls (Wahlin et al., 2013). This increase in 5hmC positive cells was seen as early as P9, and numbers were even greater at P10, prior to significant thinning of the outer nuclear layer (ONL) which occurs around 2 weeks postnatal (Wahlin et al., 2013). The authors noted that cells stained positively for either 5hmC or 5mC were also positive for the cell death terminal deoxynucleotidyl transferase dUTP nick end labelling (TUNEL) stain (Wahlin et al., 2013). The same group also investigated this phenomenon in homozygous rhodopsin-GFP knock-in mice carrying the P23H *Rho* mutation, which displayed 5mC positive cells in the early stages of degeneration. Similarly, in P23H adult retinal explants that were grown for 4 and 7 days, as the ONL degenerated there was an accumulation of 5mC positive cells, primarily in rods (Wahlin et al., 2013). These results were validated by a similar study that looked at 5mC expression in four models of RP, the *rd1* and *rd2* mouse models, which have mutations in the *Pde6b* and *Prph2* genes, respectively, and the P23H and S334ter rat models, which harbor mutations in the *Rho* gene (Farinelli et al., 2014). It was shown that at the peak of cell death in each model there was an increase in 5mC expression in photoreceptors that was colocalised with TUNEL positivity (Farinelli et al., 2014). The *rd1* mouse retina was further investigated at the ultrastructural level, revealing a severely altered chromatin structure which coincided with increased expression of the DNA methylating isozyme, DNMT3a. In microarray analysis, the *rd1* mouse showed hypermethylation of genes involved in cell death and survival, cell morphology, and nervous system development, correlating with a transcriptional silencing action. Interestingly, *rd1* retinal explants treated with the DNMT inhibitor decitabine showed a reduction in photoreceptor cell death after 4 days of treatment and a reduction in 5mC positive cells (Farinelli et al., 2014). These results suggest a potential role of DNA methylation in the pathological process of IRD and shows that DNA methylation may be a potential target for neuroprotection. However, research into this field is still in comparatively early stages, as only two studies currently have assessed the changes in DNA methylation in the context of IRD (Figure 2). As such, there is a need to understand the role of DNA methylation in the degenerative process, as well as the links between aberrant DNA methylation and photoreceptor loss in other models of IRD, and how to best translate any beneficial outcomes in preclinical research to the clinic.

## 3 Histone modifications

### 3.1 Histone acetylation and deacetylation

#### 3.1.1 The basics of histone acetylation and deacetylation

**FIGURE 3:**
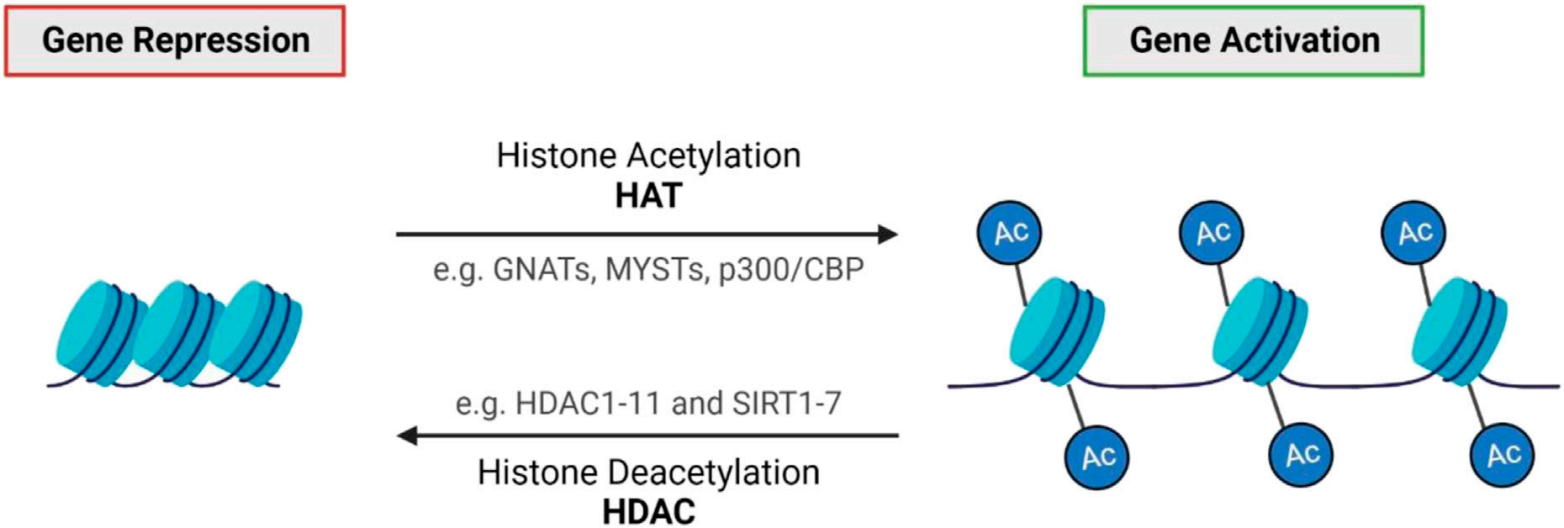
Histone acetylation and deacetylation. Histone acetylation generally results in gene activation through chromatin de-condensation, whereas deacetylation results in gene repression (Park and Kim, 2020). The balance of acetylation and deacetylation is modulated by two opposing classes of enzymes: histone acetyltransferases (HATs) and histone deacetylases (HDACs) (Bannister and Kouzarides, 2011; Park and Kim, 2020).

Histone modifications permit significant changes in the regulation of DNA and play a major role in almost all fundamental biological processes. Modifications are complex, with many chemical groups that can be added to histones such as methyl, acetyl, and ADP-ribose units (Bannister and Kouzarides, 2011). Gene expression changes vary depending on the type and location of these modifications (Bannister and Kouzarides, 2011). Post-translational acetylation and deacetylation of histone proteins allow the bidirectional regulation of gene expression and chromatin architecture by opening (acetylation) or closing (deacetylation) the chromatin structure (Park and Kim, 2020). The dynamic process and balance of acetylation and deacetylation are modulated by histone acetyltransferases (HATs) and histone deacetylases (HDAC), respectively (Figure 3) (Bannister and Kouzarides, 2011). Over the years, many studies have reported an association between altered HDAC activity and the pathology of IRDs. HDACs counteract the acetylation process modulated by HATs by removing acetyl groups from histone proteins, deacetylating histones back to their basal state, thereby suppressing gene expression (Bannister and Kouzarides, 2011). HDACs can be separated into four broad categories: Class I (HDACs 1, 2, 3, 8), Class II (HDACs 4, 5, 6, 7, 9, 10), Class III (NAD-dependent sirtuins) and Class IV (HDAC11) (Bannister and Kouzarides, 2011; Balaiya et al., 2017). Each HDAC class and its isoforms have unique biological functions, tissue specificity, enzymatic activity and more (Balaiya et al., 2017; Park and Kim, 2020). Classical HDACs (Class I, II and IV) are distinct from sirtuins (Class III HDACs), so will be discussed separately.

In the field of IRDs, research has mainly focused on establishing the role of histone deacetylation in the context of photoreceptor degeneration. A substantial decrease in acetylation (hypoacetylation) was identified in the *rd1* retina, thought to be due to an increase in HDAC class I, II, and IV activity (Sancho-Pelluz et al., 2010). Interestingly, approximately 94% of hypoacetylated cells were positive for TUNEL staining, while increased HDAC activity was detected 2 days before TUNEL positivity, suggesting that HDAC activity may precede the final stages of cell death (Sancho-Pelluz et al., 2010). This was further confirmed when *rd1* explants were treated with the pan-HDAC inhibitor, trichostatin A (TSA), which caused a significant reduction in TUNEL-positive cells. However, when treated with the class I HDAC inhibitor, Scriptaid, no neuroprotective effects on photoreceptor survival were reported (Sancho-Pelluz et al., 2010). A later study went on to identify a potential causative role of HDAC in photoreceptor degeneration, highlighting that HDAC overactivity was a common feature in ten animal models of IRD: *rd1*, *rd10*, *rd2, Cngb1* ^*−/−*^, *Rho* ^*−/−*^, S334ter, P23H, *Pde6c* ^*cpfl1*^, *Cnga3* ^*−/−*^, and *Rpe65* ^*−/−*^ (Arango-Gonzalez et al., 2014).

#### 3.1.2 Pan-HDAC inhibitors for the treatment of IRD

Due to the identification of HDAC overactivity in multiple models of IRD, many studies have searched for neuroprotective effects of pharmacological inhibition of HDACs. In the *Pde6c* ^*cpfl1*^ achromatopsia mouse model, treatment with TSA at P14, the time of onset of cone photoreceptor death in this model, resulted in cone rescue up to 10 days post-treatment (Trifunović et al., 2016). This study also showed improved localisation of cone-specific proteins, including opsins and cone transducin (GNAT2), and improved cone developmental migration patterns (Trifunović et al., 2016). When TSA was administered later in the disease stage at P18 the drug still displayed neuroprotective abilities, with a 10% increase in cone numbers and improved cone migration persisting as long as 12 days following a single intravitreal injection (Samardzija et al., 2019). TSA has also shown neuroprotective ability in *rd10* retinal explants, with a five-fold increase in surviving photoreceptors (Trifunović et al., 2018). Administration of TSA in the *rd1* and *rd10* models at later stages of the disease, P19 and P42, respectively, was sufficient to preserve and support cone survival long-term while also allowing cones to remain light sensitive with preservation of visual function (Samardzija et al., 2020). Another pan-HDAC inhibitor, SAHA, was tested in 661W cells that were stressed with a non-specific phosphodiesterase inhibitor, resulting in improved cell survival, mitochondrial respiration and reduced mitochondrial fission in the 661W cells (Perron et al., 2021). When *rd1* explants were treated with SAHA, the number of photoreceptors approximately doubled compared to controls (Perron et al., 2021; Dong et al., 2023).

Despite evidence that pharmacological HDAC inhibition is neuroprotective in several models of IRD, the molecular basis for this neuroprotection is poorly understood, mainly because HDAC inhibition drives concurrent transcriptional changes in numerous genes. For example, Samardzija et al. (2021) performed RNA sequencing analysis on *rd1* cones treated with TSA, showing that TSA may have a multi-level protection mechanism via regulation of different pro-survival pathways including MAPK, PI3K-Akt and autophagy (Samardzija et al., 2020). These studies and others have highlighted the complexity of HDAC and the impact of its inhibition. As such, more broad transcriptional studies are required to help understand the mechanisms behind the neuroprotection that arises from HDAC inhibition.

#### 3.1.3 Valproic acid and its controversial clinical translation

Only one HDAC inhibitor has been tested in clinical trials for use in RP, valproic acid (VPA); however, it sparked much debate due to highly variable patient responses and concerns raised about the study design. VPA was already FDA-approved for use in epilepsy, bipolar and migraine disorders. As previous work in animal models of RP showed VPA could inhibit apoptosis, activate microglia and stimulate photoreceptor regeneration from glial cells, drug repurposing was suggested for its therapeutic use in RP (Clemson, 2010). Additionally, VPA was found to be a potent molecular chaperone with the ability to increase the yield of properly folded mutant rhodopsin in the *Rho* ^P23H/+^ heterozygous knock-in mouse (Clemson, 2010; Kaushal et al., 2010). The initial human study reported that VPA had improved visual acuity in 9 of the 13 eyes from patients with RP; however, this study was criticised for a number of reasons, including a lack of controls and the failure to properly account for side effects from VPA use (Clemson et al., 2011; Sandberg et al., 2011). VPA was tested in a further three patients, but the trial was ended prematurely as the patients experienced a reduction in visual acuity and significant side effects including intolerable photophobia in one patient and torsional nystagmus in another, both of which were resolved upon cessation of VPA (Sisk, 2012). A subsequent non-randomised trial with ten patients showed an improved mean visual acuity after 3 months of daily VPA oral dosing, with average visual acuity progressing from 20/72 to 20/65 (Shanmugam et al., 2012). Similarly, a fourth study reported that 14 out of 15 RP patients treated with VPA had improved visual acuity (Kumar et al., 2014). Iraha et al. (2016) reported that after 6 months of VPA use, 16 out of 29 patients considered it “easier to see” when undergoing the Humphrey field analyser central 10–2 program. Patients showed improved best corrected visual acuity and visual field testing after treatment, but this improvement was lost once VPA administration was ceased (Iraha et al., 2016). Conversely, Bhalla et al. reported that in 31 patients with a range of different IRDs, there was, on average, a reduction in their visual field after VPA treatment, with most patients experiencing either no change or a slight decrease in visual acuity (Bhalla et al., 2013). Finally, a trial using VPA for 6–12 months on RP patients of unknown genotype, found no improvement in best corrected visual acuity measurements or visual field analyses, while noting potential decreases in some ERG measurement parameters (Totan et al., 2017).

Sisk (2012) suggested that genotype differences may be responsible for the variable patient outcomes (Sisk, 2012), a proposal validated by several studies conducted in animal models. A study conducted in four *Xenopus laevis* models, which expressed different RP-linked alleles of human rhodopsin, showed that administration of VPA in a *Xenopus* line with the P23H rhodopsin mutation was neuroprotective and led to an improvement in visual function (Vent-Schmidt et al., 2017). The other three *Xenopus* lines carrying the Q344ter, T17M, or T4K rhodopsin mutations did not demonstrate these same improvements (Vent-Schmidt et al., 2017). Similarly, a study carried out in two mouse models of autosomal recessive RP, the *rd1* and *rd10* mouse models, showed that daily injections for 12 days of VPA in *rd1* mice resulted in a significant increase in photoreceptor rows, with several extra rows of rod nuclei compared to PBS injected controls (Mitton et al., 2014). On the other hand, when VPA was administered in the *rd10* mouse model there was a failure of photoreceptor rescue and reduced visual function (Mitton et al., 2014). In 2018, the results of a randomised phase 2 multicentre placebo-controlled clinical trial of 90 patients with genetically characterised autosomal dominant RP revealed a small but significantly worse outcome for VPA-treated patients (Birch et al., 2018). Most adverse events reported were mild, but ultimately, the use of VPA in autosomal dominant RP was not supported (Birch et al., 2018). Future clinical translation and research of VPA or other HDAC inhibitors should consider the genotypes and clinical diagnosis of the patient and how that could affect their response to treatment with HDAC inhibitors. Importantly, when considering treatment regimes, different pan-HDAC inhibitors may have slightly different HDAC targets or have stronger affinities to certain isoforms, thus not all HDAC inhibitors will necessarily have the same effect in patients.

#### 3.1.4 Isoform-specific HDAC inhibitors for the treatment of IRD

Isoform-specific HDAC inhibition has also been investigated, allowing for a deeper understanding of HDAC subtypes that may be associated with cell death and potentially reducing off-target toxicity sometimes associated with pan-HDAC inhibitors (Bieliauskas and Pflum, 2008; Vishwakarma et al., 2013). A study that used romidepsin, an HDAC1 and HDAC2 inhibitor, in the *rd10* mouse, found that it caused significant neuroprotection and preservation of the rods, the ONL thickness increasing by approximately three-fold (Popova et al., 2021). Of concern, romidepsin also caused a reduction in weight gain throughout the treatment when compared to age-matched controls (Popova et al., 2021). In one study, no increase in cell survival was observed when the HDAC6 specific inhibitor, Tubastatin A, was applied to 661W cells that had been stressed with a non-specific phosphodiesterase inhibitor (Perron et al., 2021). Contrastingly, when 661W cells were stressed with hydrogen peroxide, treatment with Tubastatin A promoted cell survival, perhaps due to upregulation of heatshock proteins 25 and 70, heat shock transcription factor 1 and peroxiredoxin 1 (Leyk et al., 2017). Tubastatin A was then tested in the *dye* ^*ucd6*^ zebrafish model of IRD, resulting in improved retinal morphology, as assessed by qualitative improvement of the photoreceptors, a slight improvement in outer segment length, and rescue of visual function (Leyk et al., 2017). The authors suggested that HDAC6 inhibition and the associated regulation of peroxiredoxin may play a role in protecting the photoreceptors in this model (Leyk et al., 2017). In the *atp6v0e1* ^*−/−*^ zebrafish model, HDAC6 inhibition with Tubastatin A led to improved visual function and cell morphology, the treated zebrafish showing an eight-fold improvement in vision and a 44.7% improvement in photoreceptor outer segment area (Sundaramurthi et al., 2020). Proteome sequencing after treatment revealed modulation of ubiquitin-proteasome, phototransduction, metabolism, and phagosome pathways. In addition, when using *rd10* retinal explants, there was an increased number of cone arrestin-positive cells after treatment with Tubastatin A (Sundaramurthi et al., 2020). Another study used electroporation to overexpress HDAC4 to investigate its role in the degenerative process in newborn *rd1* mice (Chen and Cepko, 2009). Retinae transfected to overexpress HDAC4 (but not HDAC5 or HDAC6) contained more rods at P50, at a time when these photoreceptors would usually have degenerated (Chen and Cepko, 2009). Furthermore, compared to the full-length HDAC4 protein, expression of a short N-terminal domain of HDAC4 resulted in a more extensive preservation of *rd1* rods, greater cone survival and partial restoration of cone visual function (Guo et al., 2015). The authors speculated HDAC4s photoreceptor protection ability might be due to a restoration of altered gene expression of cell cycle progression genes *Ccnb1* and *Ccnd1*, the transcription factors c-fos, c-jun, and p53, endoplasmic reticulum stress genes such as *Atf4*, *Chop* and *Casp12* and apoptotic/cell death genes such as *Bid* and *Parp1* (Guo et al., 2015).

**FIGURE 4:**
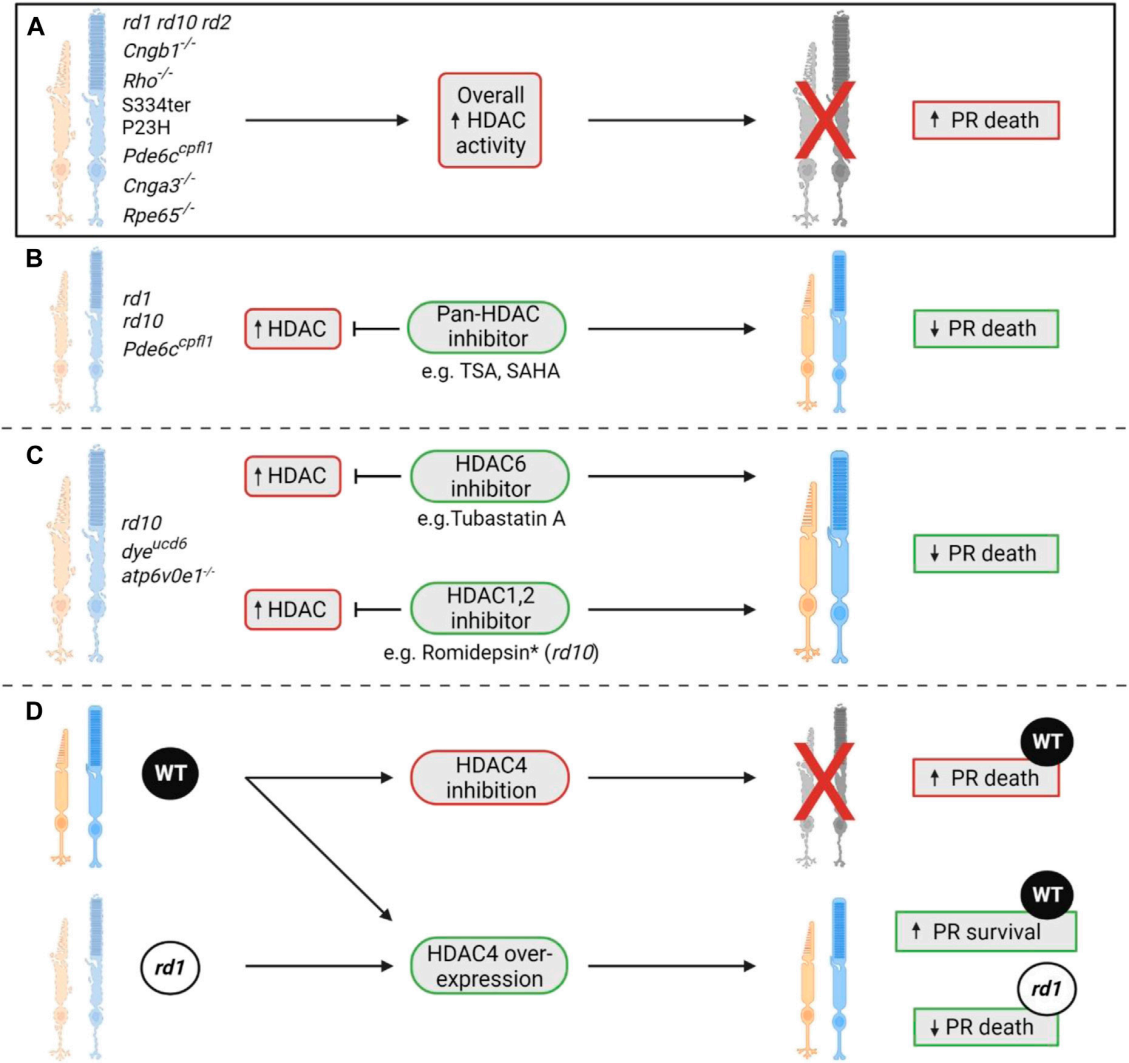
The role of HDACs in photoreceptor degeneration. **(A)** A seminal study showcased that histone deacetylase (HDAC) overactivity was a consistent phenomenon observed in ten different rodent models of IRD, namely the *rd1*, *rd10*, *rd2*, *Cngb1* ^*−/−*^, *Rho* ^*−/−*^, S334ter, P23H, *Pde6c* ^*cpfl1*^, *Cnga3* ^*−/−*^, *and Rpe65* ^*−/−*^ (Sancho-Pelluz et al., 2010; Arango-Gonzalez et al., 2014). This increase in overall HDAC activity coincided with the peak of cell death in each of these models (Sancho-Pelluz et al., 2010; Arango-Gonzalez et al., 2014). **(B)** Previous studies have shown that inhibition of HDAC with a pan-HDAC inhibitor such as trichostatin A (TSA) or SAHA can result in significant retention of photoreceptor numbers. These neuroprotective effects were displayed in two RP models (*rd1* and *rd10*) and one achromatopsia model (*Pde6c* ^*cpfl1*^) (Trifunović et al., 2016; Trifunović et al., 2018; Samardzija et al., 2019; Samardzija et al., 2020; Perron et al., 2021; Dong et al., 2023). **(C)** Isoform-specific HDAC inhibition has also proven beneficial in various models of IRD, with treatment with the HDAC1/2 inhibitor romidepsin allowing for preservation of rod numbers in the *rd10* model of RP (Popova et al., 2021). *Despite romidepsin having neuroprotective effects in the retina, it caused a reduction in weight gain throughout treatment compared to age-matched wildtype controls, displaying a potential systemic toxicity (Popova et al., 2021). HDAC6 inhibition has also been shown to be neuroprotective in the *dye* ^*ucd6*^, *atp6v0e1* ^*−/−*^ zebrafish models of inherited blindness and the *rd10* mouse model of RP (Leyk et al., 2017; Sundaramurthi et al., 2020). Each model had HDAC6 inhibited via administration of the HDAC6 inhibitor Tubastatin A. In the two zebrafish models, improvements in retinal morphology and visual function were observed (Leyk et al., 2017; Sundaramurthi et al., 2020). In the *rd10* model, an improvement in the number of cone arrestin positive cells was observed (Sundaramurthi et al., 2020). **(D)** HDAC4 inhibition in wildtype (WT) mice has been shown to cause photoreceptor death, implying that overexpression of HDAC4 is in fact neuroprotective (Chen and Cepko, 2009; Guo et al., 2015). This was validated when HDAC4 overexpression in WT and *rd1* mice showed increased photoreceptor survival in both lines (Chen and Cepko, 2009; Guo et al., 2015). PR = photoreceptor.

In summary, HDAC overactivity seems to be a consistent feature in many preclinical models of IRD, with HDAC inhibition being neuroprotective. More recently, isoform-specific studies have highlighted that not all HDAC overactivity is necessarily deleterious, with evidence that HDAC4 can be neuroprotective. Further studies should validate if such results are consistent across different models of IRD, as well as looking at HDAC isoforms that have not been investigated yet. A summary of all studies that investigate HDAC changes and consequent HDAC modulation is shown in Figure 4.

#### 3.1.5 Sirtuins–function in IRDs

The Class III HDACs, sirtuins, are a unique and highly conserved family of nicotinamide adenine dinucleotide (NAD)-dependent protein deacetylases. They deacetylate both histone and non-histone proteins and are involved in cellular functions such as stress response, apoptosis, DNA repair, cell differentiation and much more (Balaiya et al., 2017). Seven sirtuins have been identified in mammals (SIRT1-7) (Balaiya et al., 2017). The role of sirtuins was investigated in the *rd1* mouse, to ascertain if the overactive HDAC activity was derived from classical HDACs, sirtuins or both (Sancho-Pelluz et al., 2010). In the *rd1* retina, while there was a small increase in overall sirtuin activity compared to wildtype controls, classic HDACs showed a much more substantial increase (Sancho-Pelluz et al., 2010). To further elucidate if sirtuins contributed to *rd1* retinal pathology, the sirtuin inhibitor nicotinamide was administered to *rd1* explants, but no improvement in photoreceptor survival was observed (Sancho-Pelluz et al., 2010). To elucidate which specific sirtuin isoforms might be important in photoreceptor degeneration, Sirt1 immunoreactivity was assessed in retinae from *rd10* mice aged from P14 until 5 months of age (Jaliffa et al., 2009). There was strong Sirt1 staining at P15 in scattered cells throughout the ONL of the central retina (Jaliffa et al., 2009). Over time, Sirt1 immunoreactivity decreased as the *rd10* retina degenerated, following an apparent central-to-periphery gradient (Jaliffa et al., 2009). This staining was seen mostly, if not exclusively, in the nucleus of the photoreceptors, and approximately 85% of the Sirt1-positive cells were also TUNEL-positive (Jaliffa et al., 2009). Additionally, of the Sirt1-positive cells, 82% were also positive for the apoptotic marker caspase-12, and 71% for mitochondrial apoptosis inducing factor, Aif (Jaliffa et al., 2009). In a different IRD model, the *Nmnat1* ^V9M/V9M^ mutant mouse, sirtuin expression changes were assessed indirectly by examining the sites they deacetylate, such as H3K9, H3K18, and H4K16 (Greenwald et al., 2021). H3K9ac is deacetylated by Sirt1 and potentially Sirt6, H3K18ac by Sirt7, and H4K16ac, by Sirt1, Sirt2, and possibly Sirt6 (Greenwald et al., 2021). All three sites showed no significant changes compared to wildtype, suggesting that these particular sirtuins were not dysregulated as a part of *Nmnat1* ^V9M/V9M^ disease progression (Greenwald et al., 2021). Overall, only one study by Jaliffa et al. (2009) has identified sirtuin expression changes might be relevant in the degeneration seen in models of IRD (Jaliffa et al., 2009). Clearly, more work needs to be done to understand the potential role of sirtuins in different IRDs, especially since sirtuin changes have been noted in several different neurodegenerative disorders such as Alzheimer’s and Parkinson’s disease (Chandramowlishwaran et al., 2020). Increasing online access to single-cell sequencing data will permit more detailed and potentially revealing information about sirtuin expression in disease photoreceptors.

### 3.2 Histone methylation

**FIGURE 5:**
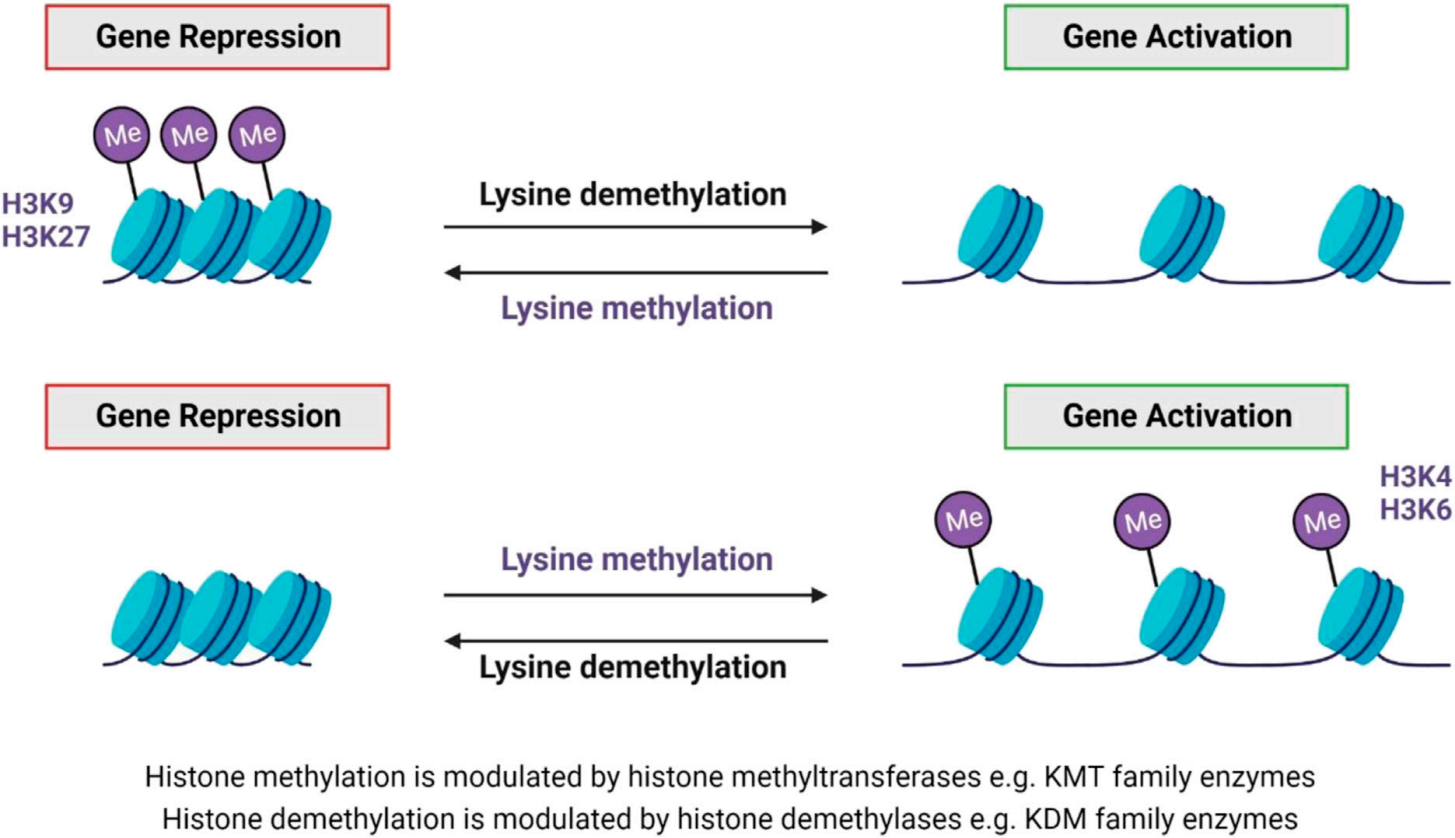
Lysine methylation and demethylation. Histone methytransferases modulate histone methylation, while demethylation is modulated by histone demethylases (Greer and Shi, 2012; Song et al., 2016). Both lysine methylation and demethylation can result in either gene repression or activation - dependent on the lysine residue that is methylated, e.g. H3K9 and H3K27 methylation results in gene repression, whereas H3K4 and H3K6 methylation result in gene activation (Basavarajappa and Subbanna, 2021).

Histone methylation and demethylation are the processes whereby methyl groups are added or removed from histone proteins (Greer and Shi, 2012). The methylation process is dynamic and supported by various enzymes, which can add or remove methyl groups on different histone types, as well as specific residues on those histones (Figure 5).

**FIGURE 6:**
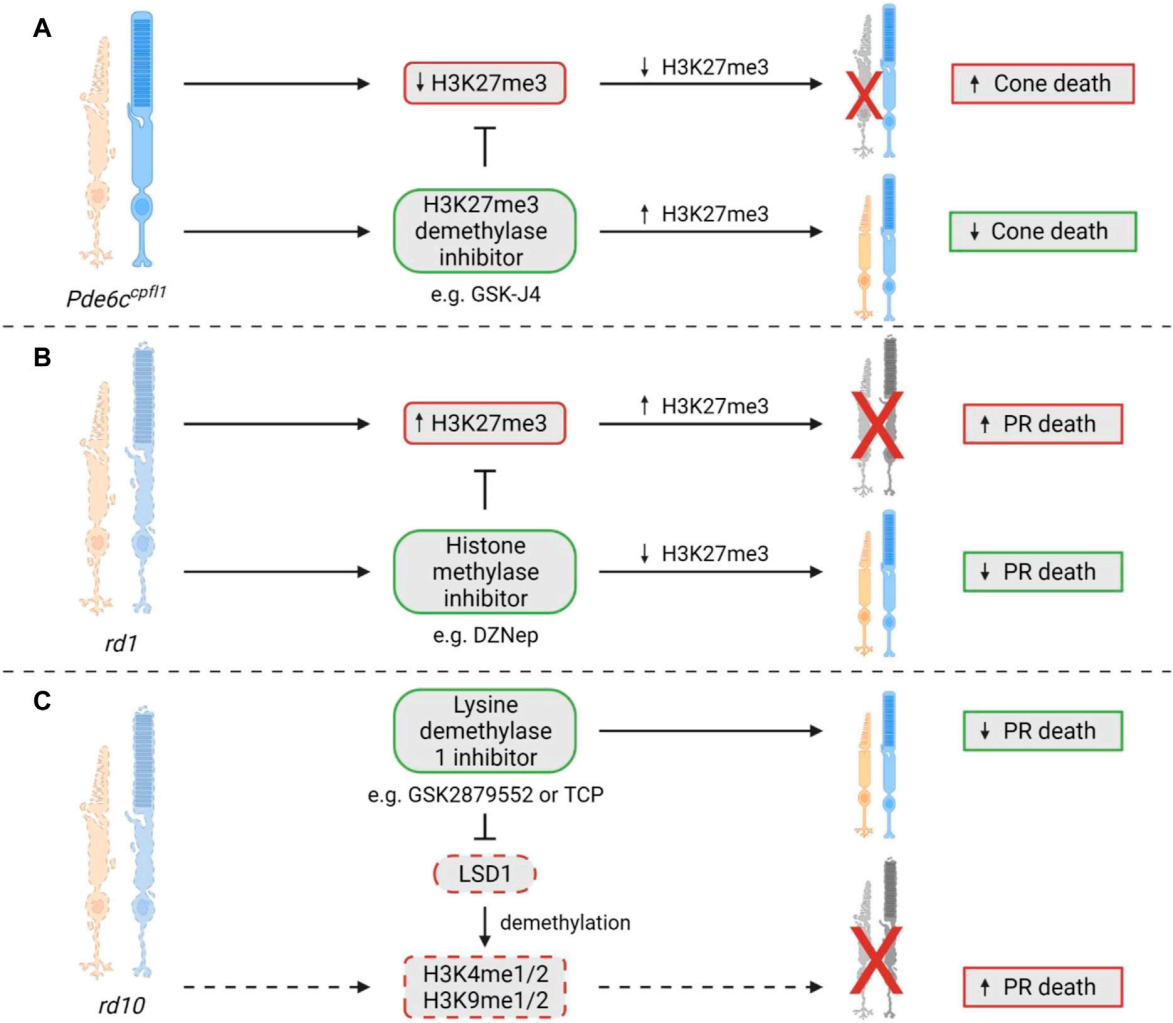
Histone methylation changes in IRDs. **(A)** In the *Pde6c* ^*cpfl1*^ mouse model of achromatopsia, decreased expression of the usually ubiquitous H3K27me3 was noted in cone photoreceptors specifically. A significant cone photoreceptor survival was observed when H3K27me3 demethylation was inhibited via GSK-J4 administration in *Pde6c* ^*cpfl1*^ retinal explants (Miller et al., 2022). **(B)** Interestingly, the *rd1* mouse model, which shows degeneration of both rod and cone photoreceptors, showed an increase in H3K27me3 expression that coincides with photoreceptor cell death. After treatment with the histone methylase inhibitor DZNep, there was a significant improvement in photoreceptor survival (Zheng et al., 2018). **(C)** A study in the *rd10* model of RP showed that treatment with a lysine demethylase 1 (LSD1) inhibitor, such as GSK2879552 or TCP, could cause a reduction in photoreceptor cell death. LSD1 is known to demethylate H3K4me1/2 and H3K9me1/2, which may contribute to cell death in this model, although the study did not directly assess this, so it remains to be confirmed (Popova et al., 2021). PR = photoreceptor.

Significantly, abnormal changes to these methylation marks have been associated with a multitude of diseases, including cancer and neurodegenerative disease (Song et al., 2016; Basavarajappa and Subbanna, 2021). Some forms of IRDs have also been associated with changes in methylation, but this field is still in its comparative infancy. In 2020, Zheng and colleagues made the first discovery of the involvement of histone methylation in IRDs, reporting increased expression of H3K27me3 in retinae from *rd1* mice (Zheng et al., 2018). The global histone methylation inhibitor DZNep was administered subretinally at P0, resulting in the preservation of both the a- and b-wave in scotopic and photopic electroretinogram (ERG) recordings at P14 (Zheng et al., 2018). With the same treatment regime, ONL thickness was significantly retained by 70% compared to untreated controls (Zheng et al., 2018). Significant improvement in ONL thickness was also seen at P21 after treatment at P0; however, improvement in the ERGs was no longer present (Zheng et al., 2018). Another study in the *rd10* model of RP reported that inhibition of the histone methylation eraser, lysine demethylase 1, LSD1, which specifically demethylates H3K4me1/2 and H3K9me1/2, resulted in reduced rod degeneration, preservation of vision, and influenced the expression of multiple genes including maintenance of rod-specific transcripts and downregulation of genes involved in inflammation, gliosis and cell death (Popova et al., 2021). The authors suggested that the neuroprotective activity of LSD1 inhibitors firstly targeted histone modifications, increasing accessibility of chromatin and upregulation of neuroprotective genes, then potentially inhibited transcription of inflammatory genes (Popova et al., 2021). Finally, in a recent study, we found that the ubiquitous H3K27me3 expression seen in wildtype cones was lost in the *Pde6c* ^*cpfl1*^ mouse model of achromatopsia (Miller et al., 2022). Administration of GSK-J4, a histone demethylase inhibitor that targets H3K27me3, resulted in increased immunostaining of H3K27me3 in *Pde6c* ^*cpfl1*^ cones, and increased cone survival in retinal explants. When GSK-J4 was administered to mice via a single intravitreal injection, there were significant transcriptional changes to pathways involved in mitochondrial dysfunction, endoplasmic reticulum stress and key epigenetic pathways (Miller et al., 2022). The role of histone methylation modifications and their contribution to IRD pathology has only recently been investigated, with current studies showing crucial differences in H3K27me3 status in cone and rod photoreceptors, where ubiquitous expression in rods is deleterious to survival, while it is beneficial in cones. A summary of all studies that have assessed changes in histone methylation in preclinical IRD models can be found in Figure 6. Future studies should investigate the differences between histone methylation patterns in rods versus cones and attempt to understand which changes to histone methylation sites are most relevant.

### 3.3 Poly(ADP-ribosyl)ation and associated processes

#### 3.3.1 The role of PARP

**FIGURE 7:**
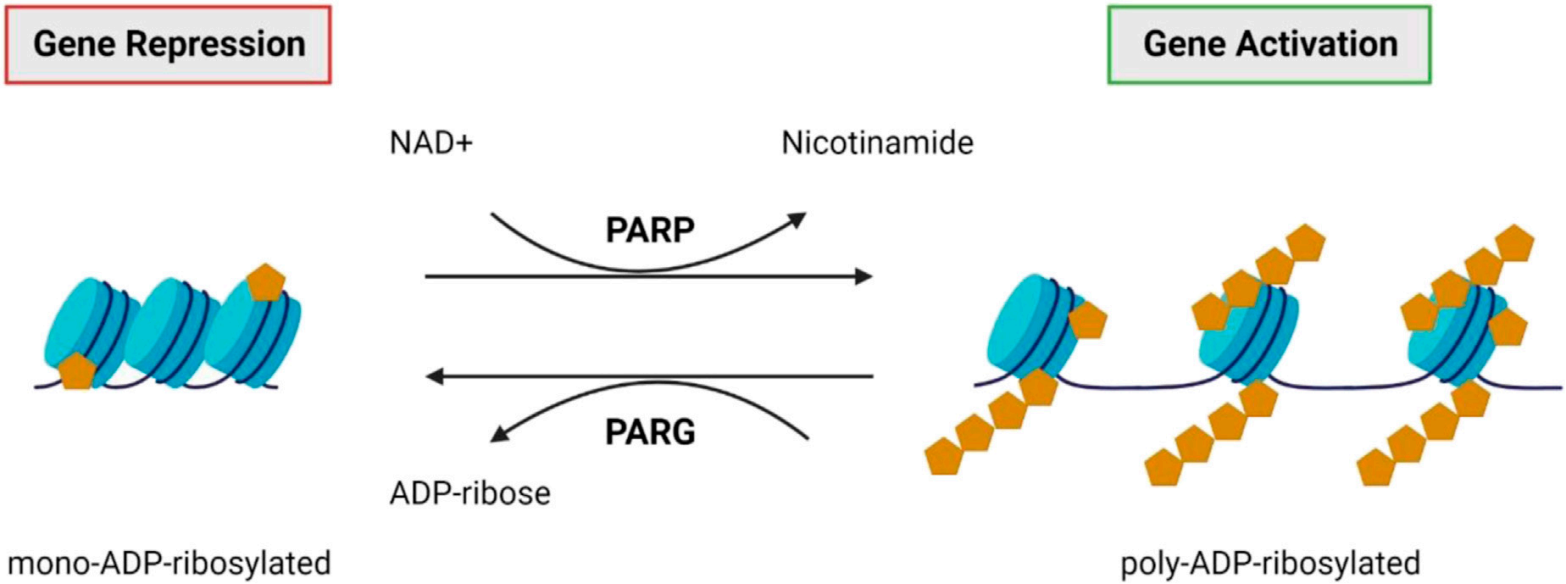
PARP and PARG. Poly (ADP-ribose) polymerases (PARPs) catalyse the attachment of poly-ADP-ribose units on the glutamic or aspartic acid residues of the target protein (Tong et al., 2001; Kraus and Lis, 2003; Quénet et al., 2009). Generally, this results in transcriptional activation via chromatin de-condensation and altered promoter and enhancer activity (Kraus and Lis, 2003; Quénet et al., 2009). This reaction is reversible by poly (ADP-ribose) glycohydrolase (PARG) activity (Quénet et al., 2009).

Poly(ADP-ribosyl)ation is a post-translational modification involving the addition of ADP-ribose units on the glutamic or aspartic acid residues of histone and non-histone target proteins, catalysed by poly (ADP-ribose) polymerase (PARP; Figure 7) (Tong et al., 2001; Kraus and Lis, 2003; Quénet et al., 2009). Modifications can involve mono ADP-ribose additions or can involve chains of ADP-ribose polymers being added, called poly (ADP-ribose) (PAR) accumulation (Langelier et al., 2018). PAR accumulation generally causes transcriptional activation via chromatin de-condensation and the alteration of promoter and enhancer activity (Kraus and Lis, 2003; Quénet et al., 2009). This reaction is reversible due to the endo- and exo-glycosidic activity of poly (ADP-ribose) glycohydrolase (PARG) (Quénet et al., 2009). PARP is involved in various cellular roles, including cell proliferation, cell death, DNA repair, genomic stability, and epigenetic regulation (Tong et al., 2001; Quénet et al., 2009). The role of poly(ADP-ribosyl)ation, and the relevant molecules in this process have garnered attention for their potential role in neurodegeneration, including in IRDs.

#### 3.3.2 PARPs in IRDs

Paquet-Durand et al. (2007) first suggested that excessive activation of PARP may have a role in the photoreceptor death seen in *rd1* mice (Paquet-Durand et al., 2007). As photoreceptors degenerated in *rd1* retinae, there was a concomitant increase in PAR positive staining which was identified via immunohistochemistry, and increased PARP activity (Paquet-Durand et al., 2007). Interestingly, in P11 *rd1* sections, 88% of PAR- or PARP-positive cells were also positive for the TUNEL cell death marker. Additionally, PAR- or PARP-positive cells were shown to co-localise with avidin and AIF, an oxidative damage marker and mitochondrial apoptosis-inducing factor, respectively (Paquet-Durand et al., 2007). The role of PARP activation in cell death was also established in two rat models of autosomal dominant RP with different mutations in the rhodopsin gene, P23H and S334ter. A significant activation of PARP was seen during cell death in these two models, which coincided with increased cellular oxidative stress, the activation of calpain, a protein linked to both apoptotic and necrotic cell death processes, and a reduction in its endogenous inhibitor calpastatin (Kaur et al., 2011). Another study looked at the impact of PARP in retinal degeneration using a *Parp1* knockout (*Parp1* ^*−/−*^) (Sahaboglu et al., 2010). The retina of the *Parp1* ^*−/−*^ mouse line was found to be morphologically similar to wildtype; however, there was a significant resistance to retinal degeneration when induced by blocking phosphodiesterase 6 (PDE6), an essential component of the phototransduction pathway. In contrast, application of the same PDE6 blocker caused rapid retinal degeneration in wildtype controls (Sahaboglu et al., 2010). The observed resistance to PDE6 induced retinal degeneration in *Parp1* ^*−/−*^ mutants suggests that PARP1 may be involved in photoreceptor degeneration via PARP-mediated cell death or a closely related mechanism (Sahaboglu et al., 2010). The role of PARP1 is largely opposed by its functional antagonist, poly-ADP-glycohydrolase (PARG), and another study by the same group investigated its effect in a *Parg110* knockout (*Parg110* ^*−/−*^) mouse. *Parg110* ^*−/−*^ mice were morphologically and functionally indistinguishable from wildtype mice, and when *Parg110* ^*−/−*^ mice were exposed to the PDE6 inhibitor there was a significant resistance to treatment, similar to that seen in *Parp1* ^−/−^ mice (Sahaboglu et al., 2014). The authors postulated that this resistance was due to low levels of PARP activity and reduced PAR accumulation, suggesting a positive regulation of PARP1 that must usually be present but is absent in the *Parg110* ^*−/−*^ retinae (Sahaboglu et al., 2014). Despite the initial assessment of PARG110 as a functional antagonist, this study revealed that there is, in fact, a positive feedback loop between PARP1 and PARG110, which is thought to be especially active in pathological conditions (Sahaboglu et al., 2014).

#### 3.3.3 Is PARP overexpression or activation a consistent finding in IRDs?

More broadly, PARP overactivity was consistently elevated compared to wildtype controls in ten models of IRD, namely the P23H and S334ter rat models of autosomal dominant RP, *rd1*, *rd2*, *rd10*, *Cngb1* ^*−/−*^ and *Rho* ^*−/−*^ mice models of autosomal recessive RP, the *Rpe65* ^*−/−*^ model of Leber’s congenital amaurosis and the *Pde6c* ^*cpfl1*^ and *Cnga3* ^*−/−*^ models of achromatopsia (Arango-Gonzalez et al., 2014). This study also highlighted the consistent overactivity of other molecules involved in a non-apoptotic cell death pathway, including calpains, protein kinase G and HDAC. These observations suggest that similar mechanisms may modulate cell death in these ten models and may allow for generic neuroprotection using drugs that target these molecules across multiple models of IRD. Jiao et al. (2016) examined four additional models of RP, all with mutations in the *Pde6a* gene (three homozygous point mutations *Pde6a* R562W, D670G, V685M, and one compound heterozygous *Pde6a* ^V685M/R562W^). In each of the four models there appeared to be PARP overactivation and PAR accumulation which correlated with the progression of photoreceptor degeneration (Jiao et al., 2016). Interestingly, the models that possessed the most rapid photoreceptor degeneration (V685M, *Pde6a* ^V685M/R562W^) seemed to have lower levels of PARP activity. In contrast, in the slower degeneration models (R562W, D670G) there was a greater amount of PARP activity in dying cells. In the D670G mutant, the mildest form of all four models, almost 100% of PARP-positive cells were also TUNEL-positive (Jiao et al., 2016). This study also reported that pharmacological PARP inhibition using PJ34, was neuroprotective in all models to varying extents (Jiao et al., 2016). All models displayed a reduction in TUNEL-positive cells after treatment as well as an increase in the number of photoreceptor rows. There appeared to be an inverse correlation between the strength of the genetic insult and the efficacy of PJ34, with the D670G model, which has the slowest degeneration, having the best treatment effects (Jiao et al., 2016). Furthermore, addition of PJ34 to retinal explant cultures preserved the number of photoreceptor rows in all models except for the V685M at 10 days, but this effect was no longer evident by 16 days, an effect that could be due to the short-term viability of retinal explants or, perhaps, loss of treatment efficacy (Jiao et al., 2016). Similar effects of PJ34 were noted in two other mouse models of RP. There was a decrease in levels of poly(ADP-ribosyl)ation and photoreceptor cell death in *rd1* retinal explants treated with PJ34 (Paquet-Durand et al., 2007), while *rd2* explants had a reduction in photoreceptor death, decreased poly(ADP ribosyl)ation, and improved rhodopsin localisation in the outer segments of rods (Sahaboglu et al., 2017).

Interestingly, a study in the *Nmnat1* ^V9M/V9M^ mouse model of IRD, which harbours a mutation in a gene responsible for NAD+ biosynthesis, showed that PARP activity was elevated during disease progression, with increased PAR expression in the photoreceptors (Greenwald et al., 2021). As PARP is a consumer of nuclear NAD+, this finding may suggest the photoreceptors in the *Nmnat1* ^V9M/V9M^ mouse might be dying via PARthanatos. This unique cell death pathway occurs due to the overactivation of PARP and overproduction of PAR rather than through classic apoptotic pathways (Fatokun et al., 2014; Greenwald et al., 2021). This hypothesis was further validated in a subsequent study by the same group where RNA sequencing of *Nmnat1* ^V9M/V9M^ retinae at 3 weeks of age showed a significant upregulation in the expression of *Parp1*, *Parp3*, *Tiparp* (*Parp7*), *Parp9*, *Parp12*, *Zchav1* (*Parp13*), *Parp14* and *Parp16* (Brown et al., 2022). By 4 weeks of age, PARP activity was significantly increased compared to wildtype controls (Brown et al., 2022). These increases in *Parp* expression appeared to coincide with reduced NAD+ activity, increased DNA damage, and increased immune reactivity in the retina (Brown et al., 2022). Furthermore, PARP upregulation has also been linked to endoplasmic reticulum (ER) stress-mediated cell death. In a model of achromatopsia caused by a mutation in the *ATF6* gene, which is best known for its role in transducing signals related to ER stress, patient fibroblasts harbouring the *ATF6* ^Y567N/Y567N^ mutation were more sensitive to ER stress and PARP overexpression (Chiang et al., 2017; Hillary and FitzGerald, 2018). Lastly, use of a monoclonal antibody that targets TNF-alpha in *rd10* RP mice resulted in a significant reduction in photoreceptor cell death, concurrently reducing PAR content, an indirect measurement of PARP activity (Martínez-Fernández de la Cámara et al., 2015).

#### 3.3.4 The implication of PARP inhibition on photoreceptor cell death

Because dysregulated PARP activity seems to be a consistent feature during the death of photoreceptors in IRD, and the use of PJ34 to inhibit PARP appeared beneficial, multiple other PARP inhibitors have been tested to assess their effectiveness in preclinical models. These include inhibitors that are FDA-approved or in late stages of clinical trials, with the hope for easier drug repurposing in the future. R503, ABT-888 (in phase 3 clinical trials) and Olaparib (FDA-approved for use in ovarian cancer treatment) were all tested for their effectiveness in *rd1* mice, with R503 and ABT-888 showing relative toxicity at low drug concentrations (Sahaboglu et al., 2016). Contrastingly, the FDA-approved Olaparib, which targets PARP1 and PARP2 isoforms, did not show toxicity and exhibited photoreceptor protection after treatment, in both short-term (treatment starting at P7 and finishing at P11) and long-term experiments (P7-P17). Olaparib reduced the number of TUNEL-positive cells and decreased PARylation while preserving ONL thickness (Sahaboglu et al., 2016). There was also a reduction in cGMP levels, thought to be an essential component of cell death in this model (Sahaboglu et al., 2016). However, this neuroprotective effect was lost by P24 (Sahaboglu et al., 2016). In a separate study, another two PARP inhibitors, BMN-673 (FDA-approved) and 3-aminobenzamine were utilised in the *rd1* mouse, and both were able to reduce photoreceptor cell death by 25%–40%. The authors suggested this survival may be due to a relationship between PARP and the highly conserved kinase GSK and Wnt/catenin pathways, which are involved in various cellular processes such as differentiation, adult tissue homeostasis and apoptosis (Antolín and Mestres, 2014; Yang et al., 2016; Pai et al., 2017; Sahaboglu et al., 2020). Before treatment, there was a reduction in GSK-alpha immunoreactivity in *rd1* retinae in the ganglion cell and inner cell layers, and a small but not significant reduction in the ONL. When treated with the PARP inhibitors, these expression levels were reversed towards wildtype levels. Beta-catenin showed a significantly lower expression in the RPE, but no significant reduction in the ganglion cell layer and inner nuclear layer. These changes were partially neutralised by BMN-673 in the ganglion cell layer and the RPE, and by 3-aminobenzamide in the ganglion cell layer, RPE and the inner nuclear layer (Sahaboglu et al., 2020).

**FIGURE 8:**
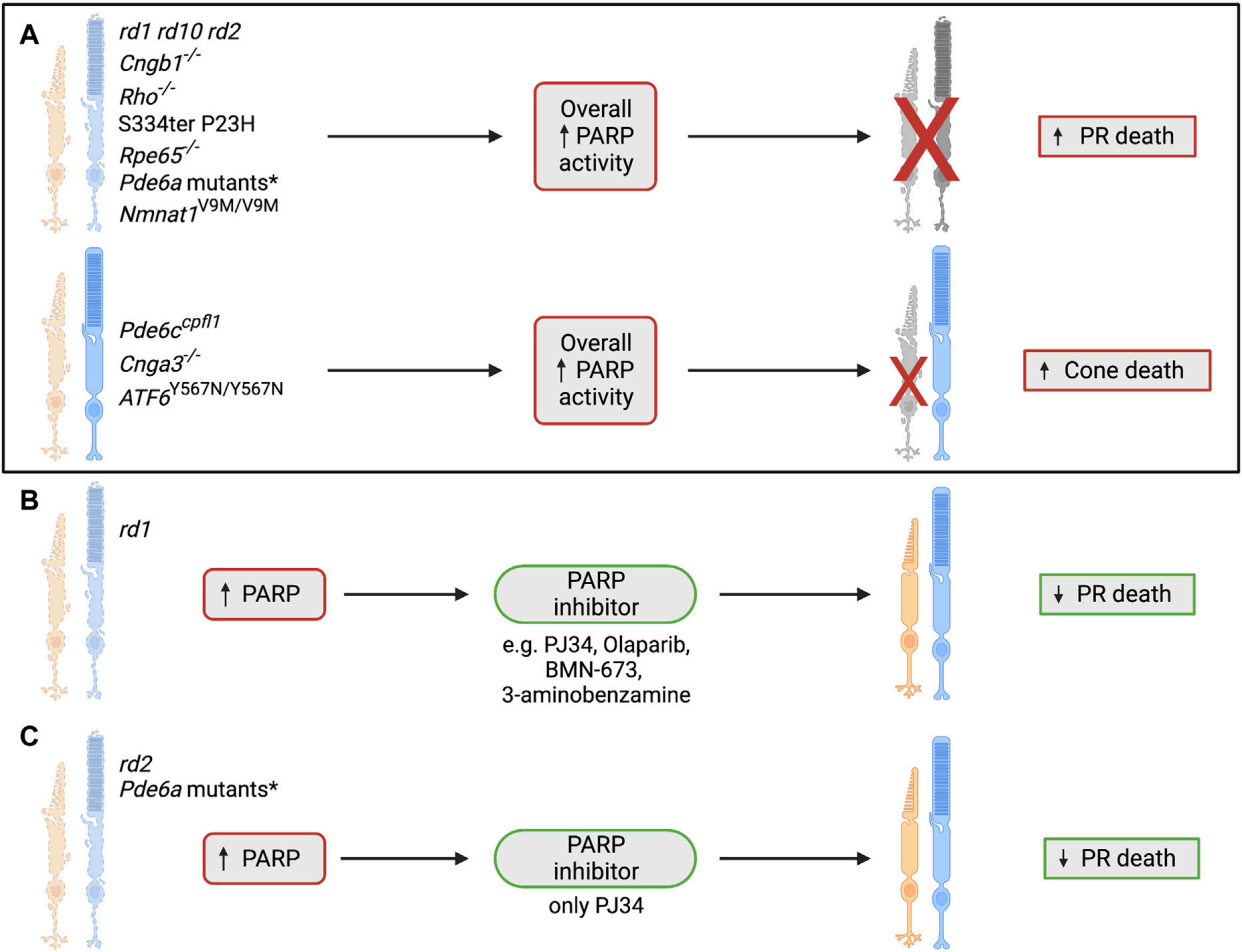
The role of poly(ADP-ribosyl)ation and PARP in IRDs. **(A)** PARP overactivity has previously been shown in many IRD models, including autosomal dominant RP, autosomal recessive RP, Leber’s congenital amaurosis, cone/rod dystrophy and achromatopsia. This consistent overactivity of PARP has been shown to coincide with photoreceptor cell death, suggesting a link between these two processes (Paquet-Durand et al., 2007; Kaur et al., 2011; Arango-Gonzalez et al., 2014; Jiao et al., 2016; Chiang et al., 2017; Greenwald et al., 2021). *The *Pde6a* mutant models that have shown an increase in overall PARP activity are *Pde6a* R562W, *Pde6a* D670G, *Pde6a* V685M and *Pde6a* ^V685M/R562W^ (Jiao et al., 2016). **(B)** Testing of several PARP inhibitors has taken place in the *rd1* model and has indicated PARP inhibition as a strong candidate for neuroprotection of photoreceptors in IRD. Photoreceptor survival has been noted after administration of PJ34, Olaparib, BMN-673, and 3-aminobenzamine (Paquet-Durand et al., 2007; Sahaboglu et al., 2016; Sahaboglu et al., 2020). **(C)** Various IRD models have been used to test the PARP inhibitor PJ34, including the *rd1*, *rd2*, *Pde6a* R562W, *Pde6a* D670G, *Pde6a* V685M and *Pde6a* ^V685M/R562W^ models. PJ34 has shown neuroprotective benefits in all mentioned models and reduces photoreceptor death after administration (Paquet-Durand et al., 2007; Jiao et al., 2016; Sahaboglu et al., 2017). *The *Pde6a* mutant models that have shown a reduction in photoreceptor death after treatment with PJ34 are *Pde6a* R562W, *Pde6a* D670G, *Pde6a* V685M and *Pde6a* ^V685M/R562W^ (Jiao et al., 2016). PR = photoreceptor.

Given the data suggesting the influence of PARP in multiple IRDs, and the fact that PARP inhibition generally enhances photoreceptor survival (summary in Figure 8), the next steps in this field should include developing a firm understanding of the mechanisms behind this protection. Analysis of PARP inhibition in clinical trials involving IRD patients should be undertaken to determine if PARP inhibitors can benefit all patients or only a small subset dependent on genotype or mutation, and determine the safety of long-term treatment and its effect on disease progression.

### 3.4 Interactions between different post-translational modifications

Post-translational modifications such as DNA methylation, histone acetylation, histone methylation and poly(ADP-ribosyl)ation all have complex interactions and functional interplay. Several studies in IRD have highlighted these complex relationships, emphasising that epigenetic modifications do not take place in isolation (Nakao, 2001; Lee et al., 2006; Ummarino et al., 2021; Khalid et al., 2022; Miller et al., 2022). In 2010, Sancho-Pelluz and colleagues discovered that both HDAC and PARP were overactive in *rd1* mice (Sancho-Pelluz et al., 2010). Interestingly, they found that HDAC overactivity preceded PARP overactivity by approximately 2 days, with these findings later validated in a separate study that observed the same pattern of HDAC overactivity preceding PARP overactivity in ten different models of IRD (Sancho-Pelluz et al., 2010; Arango-Gonzalez et al., 2014). Additionally, it was found that PARP overactivity coincided with the peak of cell death, a determination based on positive TUNEL staining (Arango-Gonzalez et al., 2014). Notably, they found that calpain and PARP overactivity coincided with TUNEL staining, indicating that they may be involved in the final stages of cell death, as TUNEL labels DNA nick-ends which are associated with final stages of cell death, while HDAC overactivity and cGMP accumulation appeared to be found earlier in the cell death process (Arango-Gonzalez et al., 2014). Dong et al. (2023) also showed that treating *rd1* explants with the PARP inhibitor Olaparib improved photoreceptor survival and reduced HDAC activity (Dong et al., 2023). In a similar line, overexpression of HDAC4 using electroporation significantly increased rod photoreceptor survival in *rd1* mice retinae (Guo et al., 2015). HDAC4 overexpression led to a 50% decrease in *Parp1* expression, along with other markers for cell death, cell cycle genes, and oxidative and endoplasmic reticulum stress, suggesting that *Parp1*, with other vital genes, may be involved in the protective effect seen with HDAC4 overexpression (Guo et al., 2015).

HDAC has also been shown to interact with other epigenetic modifications, such as histone methylation, where treatment with the pan-HDAC inhibitor TSA in the *Pde6c* ^*cpfl1*^ mouse model of achromatopsia resulted in changes to histone methylation status. H3K27me3 levels which are severely reduced in *Pde6c* ^*cpfl1*^ mice compared to wildtype, were partially restored to wildtype levels upon treatment, highlighting the effect that HDAC inhibition has on histone methylation (Miller et al., 2022). HDAC has also been shown to interact with DNA methylation via DMNT activity in *rd1* and *rd2* mice as well as in S334ter and P23H rat models of RP. Each model showed 5mC positive cells had very low or absent levels of acetylated lysine, suggesting a key interplay between HDAC and DNMT (Farinelli et al., 2014). Functional interplay between DNA methylation changes and poly(ADP-ribosyl)ation has been suggested; for instance, a study on *rd1* retinae revealed that many cells in the ONL that were positive for 5mC staining were also positive for PAR staining (Wahlin et al., 2013; Ummarino et al., 2021). Contrastingly, another study showed that PARP inhibition during the peak of degeneration in *rd1* retinae, did not cause any changes to 5mC and 5hmC levels, suggesting that DNA methylation may actually be unrelated or upstream to PARP activity (Sahaboglu et al., 2016).

The understanding of interactions between different epigenetic modifications in the context of IRD is still in relative infancy. In the future these types of studies may help us understand the neuroprotective effects of these drugs on a mechanistic level and may be used to leverage the use of multiple epigenetic modifying drugs for a synergistic and protective effect.

## 4 Conclusion

The potential role of epigenetic modifiers in IRD pathology has been gaining new insights in recent years. Roles for DNA methylation and histone modifications such as deacetylation, methylation, and poly(ADP-ribosyl)ation have been suggested, with modulation of each being a potential therapeutic target. The development of new cell-specific epigenetic techniques such as CUT&Tag, for example, will greatly assist in elucidating the role of histone modifications in IRD disease processes and its potential for therapeutic targeting. While understanding DNA methylation and histone methylation in IRD is still quite a new field, the influence of PARP and HDACs have been more extensively studied. PARP inhibition has been tested in multiple preclinical models and a better understanding of the mechanisms that underlie its neuroprotective action will only improve therapeutic options in the future. Both pan- and selective HDAC inhibition have shown promising potential in various preclinical models, although the HDAC inhibitor VPA remains the only drug that has so far proceeded to clinical trials. However, likely due to its different impact depending on the genetic basis of the IRD, its further use is currently discouraged due to inconsistent results in these clinical studies. A better understanding of how HDAC inhibitors affect people with different genotypes will facilitate future clinical translation of these types of drugs. There may also be sex differences in epigenetic regulation and drug metabolism that need to be considered (Gegenhuber and Tollkuhn, 2019; Li et al., 2019; Oliva et al., 2020; Saravanan et al., 2023). Clearly, interaction between each of these epigenetic regulators are very complex, with functional relationships via diverse molecules and intracellular pathways. In order to best understand these complex relationships, further omics studies are needed, ideally concurrently, which would allow for a better understanding of cell and mutation specific differences. Using multiple omics platforms in parallel would also allow for superior discernment of the changes underpinning the protective effects of epigenetic modulating drugs. This in-depth grasp of cellular mechanisms will be essential before successful translation of therapies to the clinic.

## Abbreviations

5caC, 5-carboxylcytosine; 5fC, 5-formylcytosine; 5hmC, 5-hydroxymethylcytosine; 5mC, 5-methylcytosine; BER, base excision repair; DNMT, DNA methyltransferase; HAT, histone acetyltransferase; HDAC, histone deacetylase; IRD, inherited retinal disease; ONL, outer nuclear layer; PAR, poly (ADP-ribose); PARG, poly (ADP-ribose) glycohydrolase; PARP, poly (ADP-ribose) polymerase; rd, retinal degeneration [mouse model]; RP, retinitis pigmentosa; TDG, thymine DNA glycosylase; TET, ten-eleven translocase; TSA, trichostatin A; TUNEL, t erminal deoxynucleotidyl transferase dUTP nick end labelling; VPA, valproic acid.
