# Supplementary material for: The role of epigenetic changes in the pathology and treatment of inherited retinal diseases
Source: Front Cell Dev Biol. 2023 Aug 4;11:1224078. doi: 10.3389/fcell.2023.1224078 (PMC10436478; doi:10.3389/fcell.2023.1224078)
Supplement: Supplementary file 1 [file Table1.docx]

Supplementary Table 1. Summary table of all preclinical models of IRD discussed in this review paper.

| Model | Species | Mutated gene name | Gene acronym | Role of mutated gene | Disease phenotype |
| --- | --- | --- | --- | --- | --- |
| *rd1* (1, 2) | Mouse | Phosphodiesterase 6B | *Pde6b* | Rod phototransduction | Autosomal recessive RP |
| *rd10* (3) | Mouse | Phosphodiesterase 6B | *Pde6b* | Rod phototransduction | Autosomal recessive RP |
| *rd2* (4) | Mouse | Peripherin 2 | *Prph2* | Structural role in photoreceptors | Autosomal recessive RP, autosomal dominant RP |
| P23H (5, 6) | Rat | Rhodopsin | *Rho* | Rod phototransduction | Autosomal dominant RP |
| S334ter (6) | Rat | Rhodopsin | *Rho* | Rod phototransduction | Autosomal dominant RP |
| *Rho*^P23H/+^ heterozygous knock-in (7) | Mouse | Rhodopsin | *Rho* | Rod phototransduction | Autosomal dominant RP |
| *Rho*^P23H/P23H^ homozygous knock-in (7) | Mouse | Rhodopsin | *Rho* | Rod phototransduction | Autosomal dominant RP |
| *Rho*^-/-^ (8) | Mouse | Rhodopsin | *Rho* | Rod phototransduction | Autosomal recessive RP |
| P23H (9) | *Xenopus laevis* | Rhodopsin | *Rho* | Rod phototransduction | Autosomal dominant RP |
| Q334ter (9) | *Xenopus laevis* | Rhodopsin | *Rho* | Rod phototransduction | Autosomal dominant RP |
| T17M (9) | *Xenopus laevis* | Rhodopsin | *Rho* | Rod phototransduction | Autosomal dominant RP |
| T4K (9) | *Xenopus laevis* | Rhodopsin | *Rho* | Rod phototransduction | Autosomal dominant RP |
| *Cngb1*^-/-^ (10) | Mouse | Cyclic nucleotide gated channel subunit beta 1 | *Cngb1* | Rod phototransduction | Autosomal recessive RP |
| *Pde6a*^R562W/R562W^ (11) | Mouse | Phosphodiesterase 6A | *Pde6a* | Rod phototransduction | Autosomal recessive RP |
| *Pde6a*^D670G/D670G^ (11) | Mouse | Phosphodiesterase 6A | *Pde6a* | Rod phototransduction | Autosomal recessive RP |
| *Pde6a*^V685M/V685M^ (11) | Mouse | Phosphodiesterase 6A | *Pde6a* | Rod phototransduction | Autosomal recessive RP |
| *Pde6a*^V685M/R562W^ (11) | Mouse | Phosphodiesterase 6A | *Pde6a* | Rod phototransduction | Autosomal recessive RP |
| *Rpe65*^-/-^ (12, 13) | Mouse | Retinal pigment epithelium-specific 65 kDa protein/retinoid isomerohydrolase | *Rpe65* | Regeneration of cone and rod visual pigments | Lebers congenital amaurosis and autosomal recessive RP |
| *dye^ucd6^* (14) | Zebrafish | ATPase H+ transporting V0 subunit e1 | *Atp6v0e1* | Enzyme transporter | Inherited blindness |
| *atp6v0e1^-/-^* (15) | Zebrafish | ATPase H+ transporting V0 subunit e1 | *Atp6v0e1* | Enzyme transporter | Inherited blindness |
| *Pde6c^cpfl1^* (16) | Mouse | Phosphodiesterase 6C | *Pde6c* | Cone phototransduction | Achromatopsia/cone dystrophy |
| *Cnga3^-/-^* (17) | Mouse | Cyclic nucleotide gated channel subunit alpha 3 | *Cnga3* | Cone phototransduction | Achromatopsia/cone dystrophy |
| *ATF6*^Y567N/Y567N^ fibroblasts (18) | Human | Activating transcription factor 6 | *ATF6* | Endoplasmic reticulum stress-regulated transmembrane transcription factor | Achromatopsia/cone dystrophy |
| *Nmnat1*^V9M/V9M^ (19) | Mouse | Nicotinamide mononucleotide adenylytranferase 1 | *Nmnat1* | Biosynthesis of NAD | Cone/cone-rod dystrophy |

**References**

1. Keeler CE. The Inheritance of a Retinal Abnormality in White Mice. Proceedings of the National Academy of Sciences of the United States of America. 1924;10(7):329-33.

2. Bowes C, Li T, Danciger M, Baxter LC, Applebury ML, Farber DB. Retinal degeneration in the rd mouse is caused by a defect in the β subunit of rod cGMP-phosphodiesterase. Nature. 1990;347(6294):677-80.

3. Gargini C, Terzibasi E, Mazzoni F, Strettoi E. Retinal organization in the retinal degeneration 10 (rd10) mutant mouse: A morphological and ERG study. Journal of Comparative Neurology. 2007;500(2):222-38.

4. Chang B, Hawes NL, Hurd RE, Davisson MT, Nusinowitz S, Heckenlively JR. Retinal degeneration mutants in the mouse. Vision Research. 2002;42(4):517-25.

5. Orhan E, Dalkara D, Neuillé M, Lechauve C, Michiels C, Picaud S, et al. Genotypic and phenotypic characterization of P23H line 1 rat model. PLoS One. 2015;10(5):e0127319.

6. LaVail MM, Nishikawa S, Steinberg RH, Naash MI, Duncan JL, Trautmann N, et al. Phenotypic characterization of P23H and S334ter rhodopsin transgenic rat models of inherited retinal degeneration. Exp Eye Res. 2018;167:56-90.

7. Sakami S, Maeda T, Bereta G, Okano K, Golczak M, Sumaroka A, et al. Probing mechanisms of photoreceptor degeneration in a new mouse model of the common form of autosomal dominant retinitis pigmentosa due to P23H opsin mutations. The Journal of biological chemistry. 2011;286(12):10551-67.

8. Jaissle GB, May CA, Reinhard J, Kohler K, Fauser S, Lütjen–Drecoll E, et al. Evaluation of the Rhodopsin Knockout Mouse as a Model of Pure Cone Function. Investigative Ophthalmology & Visual Science. 2001;42(2):506-13.

9. Vent-Schmidt RYJ, Wen RH, Zong Z, Chiu CN, Tam BM, May CG, et al. Opposing Effects of Valproic Acid Treatment Mediated by Histone Deacetylase Inhibitor Activity in Four Transgenic X. laevis Models of Retinitis Pigmentosa. J Neurosci. 2017;37(4):1039-54.

10. Hüttl S, Michalakis S, Seeliger M, Luo DG, Acar N, Geiger H, et al. Impaired channel targeting and retinal degeneration in mice lacking the cyclic nucleotide-gated channel subunit CNGB1. J Neurosci. 2005;25(1):130-8.

11. Sothilingam V, Garcia Garrido M, Jiao K, Buena-Atienza E, Sahaboglu A, Trifunović D, et al. Retinitis pigmentosa: impact of different Pde6a point mutations on the disease phenotype. Human Molecular Genetics. 2015;24(19):5486-99.

12. Redmond TM, Yu S, Lee E, Bok D, Hamasaki D, Chen N, et al. Rpe65 is necessary for production of 11-cis-vitamin A in the retinal visual cycle. Nature Genetics. 1998;20(4):344-51.

13. Cai X, Conley SM, Naash MI. RPE65: role in the visual cycle, human retinal disease, and gene therapy. Ophthalmic Genet. 2009;30(2):57-62.

14. Daly C, Shine L, Heffernan T, Deeti S, Reynolds AL, O’Connor JJ, et al. A Brain-Derived Neurotrophic Factor Mimetic Is Sufficient to Restore Cone Photoreceptor Visual Function in an Inherited Blindness Model. Scientific Reports. 2017;7(1):11320.

15. Sundaramurthi H, Roche SL, Grice GL, Moran A, Dillion ET, Campiani G, et al. Selective Histone Deacetylase 6 Inhibitors Restore Cone Photoreceptor Vision or Outer Segment Morphology in Zebrafish and Mouse Models of Retinal Blindness. Front Cell Dev Biol. 2020;8:689.

16. Chang B, Grau T, Dangel S, Hurd R, Jurklies B, Sener EC, et al. A homologous genetic basis of the murine cpfl1 mutant and human achromatopsia linked to mutations in the PDE6C gene. Proc Natl Acad Sci U S A. 2009;106(46):19581-6.

17. Biel M, Seeliger M, Pfeifer A, Kohler K, Gerstner A, Ludwig A, et al. Selective loss of cone function in mice lacking the cyclic nucleotide-gated channel CNG3. Proceedings of the National Academy of Sciences. 1999;96(13):7553-7.

18. Chiang WC, Chan P, Wissinger B, Vincent A, Skorczyk-Werner A, Krawczyński MR, et al. Achromatopsia mutations target sequential steps of ATF6 activation. Proc Natl Acad Sci U S A. 2017;114(2):400-5.

19. Nash BM, Symes R, Goel H, Dinger ME, Bennetts B, Grigg JR, et al. NMNAT1 variants cause cone and cone-rod dystrophy. Eur J Hum Genet. 2018;26(3):428-33.
